# Separate and combined effects of cold dialysis and intradialytic exercise on the thermoregulatory responses of hemodialysis patients: a randomized-cross-over study

**DOI:** 10.1186/s12882-020-02167-z

**Published:** 2020-12-02

**Authors:** Argyro A. Krase, Andreas D. Flouris, Christina Karatzaferi, Christoforos D. Giannaki, Ioannis Stefanidis, Giorgos K. Sakkas

**Affiliations:** 1 FAME Lab, School of Physical Education, Sport Science and Dietetics, University of Thessaly, Trikala, Greece; 2 School of Physical Education, Sport Science and Dietetics, University of Thessaly, Trikala, 42100 Greece; 3 Department of Life and Health Sciences, University of Nicosia, Nicosia, Cyprus; 4 School of Health Sciences, Department of Medicine, Division of Nephrology, University of Thessaly, Larissa, Greece; 5 School of Sports and Health Sciences, Cardiff Metropolitan University, Cardiff, UK

**Keywords:** Cold dialysis, Body temperature during dialysis, Dialysis temperature, Thermal balance

## Abstract

**Background:**

The separate and combined effects of intradialytic exercise training (IET) and cold dialysis (CD) on patient thermoregulation remain unknown. This study assessed the thermoregulatory responses of hemodialysis patients under four different hemodialysis protocols: a) one typical dialysis (TD) protocol (dialysate temperature at 37 °C), b) one cold dialysis (CD) protocol (dialysate temperature at 35 °C), c) one typical dialysis protocol which included a single exercise bout (TD + E), d) one cold dialysis protocol which included a single exercise bout (CD + E).

**Methods:**

Ten hemodialysis patients (57.2 ± 14.9 years) participated in this randomized, cross-over study. Core and skin temperatures were measured using an ingestible telemetric pill and by four wireless iButtons attached on the skin, respectively. Body heat storage (S) calculated using the thermometric method proposed by Burton.

**Results:**

The TD and TD + E protocols were associated with increased S leading to moderate effect size increases in core body temperature (as high as 0.4 °C). The low temperature of the dialysate during the CD and the CD + E protocols prevented the rise in S and core temperature (*p* > 0.05), even during the period that IET took place.

**Conclusions:**

TD and IET are accompanied by a moderate level of hyperthermia, which can be offset by CD. We recommended that CD or with IET can prevent the excessive rise of S.

**Trial registration:**

Clinical Trial Registry number: NCT03905551 (clinicaltrials.gov), DOR: 05/04/2019,

## Background

Hemodialysis (HD) represents a significant challenge for the thermoregulatory system of HD patients [1, 2]. Indeed, body temperature rises during HD due to (i) heat transfer into the body via the heated dialysate, (ii) endogenous heat production through normal metabolic processes, and (iii) attenuated heat loss at the skin surface [3]. The latter has been hypothesized to occur because cutaneous vessels are constricted during typical dialysis (TD; 37 °C dialysate temperature) due to loss of blood volume towards the extracorporeal circuit [4]. This leads to attenuated heat dissipation from the skin surface despite the fact that metabolic heat production remains relatively stable [5]. Consequently, heat is accumulated (this is typically referred to as “increased body heat storage”) and, soon, core temperature rises during a typical session of hemodialysis [6]. This increased heat storage (S) offsets the vasoconstrictive response to hypovolemia [7] and it is one of the responsible contributing factor which leading to the intradialytic hypotension causing patient discomfort and increased mortality [8, 9].

Lowering the dialysate temperature (35–36 °C; cold dialysis) has been proposed as a simple and useful method to reduce heat storage during hemodialysis and, therefore, decrease the frequency of intradialytic hypotension episodes [5, 10, 11]. Indeed, cold dialysis (CD), has been proposed that attenuates the risk for patient hyperthermia compared to typical dialysis (dialysate temperature at 37 °C; TD) and leads to cutaneous vasoconstriction [12]. These observations have led to a growing interest in the thermal and circulatory adaptations occurring during CD [13]. Yet, the precise changes in body heat balance during either CD or TD remain poorly documented and understood.

The above-mentioned benefits of CD in thermoregulatory and cardiovascular stability are highlighted even further due to the well accepted adoption of intra-dialytic exercise training programs [14]. It has been well-established that intra-dialytic exercise leads to benefits in strength and endurance [14–16] as well as improved clearance (Kt/v), [17, 18] hemodynamic stability, and patient quality of life [14]. The beneficial effects of intradialytic-exercise are likely due to increased muscle blood flow and reduced inter-compartmental resistance by peripheral vasodilation [19, 20]. Overcoming this resistance seems to be the single most effective method for improved toxin removal during hemodialysis [21, 22]. Nevertheless, this vasodilation is augmented further during TD by the exercise-induced hyperthermia [19] and may, therefore, increase the frequency of intradialytic hypotension episodes. As indicated above, we hypothesized that CD may be able to minimize the need for peripheral vasodilation, leading to improved patient thermoregulatory and cardiovascular stability. However, despite a strong rationale for the implementation of intradialytic exercise training programs and the aforementioned benefits of CD, the separate and combined effects of these protocols on patient thermoregulation have not been investigated to date.

## Methods

### Aims

The current study aimed to assess the thermoregulatory responses of hemodialysis patients under four different hemodialysis (240 min) protocols: a) one typical dialysis (TD) protocol (dialysate temperature at 37 °C), b) one cold dialysis (CD) protocol (dialysate temperature at 35 °C), c) one typical dialysis protocol which included a single exercise bout (TD + E), d) one cold dialysis protocol which included a single exercise bout (CD + E).

### Study design

Ten hemodialysis patients participated in this randomized, cross-over study were recruited from a single hemodialysis unit at the General Hospital of Trikala, Thessaly, Greece. All study measurements were performed at a hospital climate control room using the metabolic ward of the General Hospital of Trikala, Greece. The mean age was 57.2 ± 14.9 years (Table 1)*.* Patients enrolled by a research assistant assigned into the study while the order that the patients assigned to the first scenario was random using a computer random number generator. Each patient was monitored during a) one protocol of typical dialysis with dialysate temperature at 37 °C (TD), b) one protocol of cold dialysis with dialysate temperature at 35 °C (CD), c) one protocol of typical dialysis which included a single exercise bout (TD + EP) and d) one protocol of cold dialysis which included a single exercise bout (CD + EP). The dialysate temperature was fixed for the whole dialysis protocol. The patient participants underwent dialysis therapy (Fresenius 4008B, Oberursel, Germany) 3 times/week with low flux, hollow fiber dialyzers (dialysis duration 4 h). The dialysis protocols were performed using dialysis flow at 550 ml/min and mean average of conductivity dialysance at 137–140 mEq/L. The net ultrafiltration weight was the same in all sessions. All patients were clinically stable and they had received regular hemodialysis treatment for at least 3 months, with adequate dialysis delivery Kt/V > 1.1 and good compliance of dialysis treatment, the serum albumin was > 2.5 g/dL, hemoglobin ≥11 g/dL and treated with rHuEPO. Patients were not eligible for participation in the study if they had a reason to be in a catabolic state, such as hyperthyroidism, active vasculitis, malignancies, pregnant, HIV, opportunistic infections, musculoskeletal contraindication to exercise, requirement for systemic anticoagulation, participant or participated in another trial within 1 month or inflammations, that required intravenous antibiotics within 3 months prior to enrollment, diabetics receiving insulin therapy, New York Heart Association grade IV heart failure, and mental incapacity to consent.

**Table 1:** Individual characteristics of the study group

Patient (n)	Age (yr) range	Dry Weight (kg)	Height (cm)	Body surface area (m^**2**^)	Cause of end-stage renal disease
1	80–85	69.7	161	1.77	Glomerulonephritis
2	50–55	73.6	175	1.90	Nephrectomy
3	60–65	69.0	185	1.88	Polycystic kidney disease
4	60–65	80.0	175	1.97	Glomerulonephritis
5	60–65	80.5	178	2.00	Glomerulonephritis
6	60–65	64.2	158	1.68	Polycystic kidney disease
7	30–35	66.0	173	1.78	IgA nephropathy
8	50–55	85.3	174	2.03	Polycystic kidney disease
9	65–70	78.8	172	1.94	Unknown causes
10	35–40	48.3	145	1.39	Glomerulosclerosis

Note: Mean ± SD; Age: 57.2 ± 14.9; Dry Weight: 71.4 ± 10.0; Height: 169.6 ± 11.6; Body Surface: 1.83 ± 0.18

Dialysis protocols were performed in a random order at the same time and day (second dialysis of the week) of the week to minimize differences in ultrafiltration volume between the four protocols and 1 week apart. The ambient temperature in the room was 25.2–25.9 °C. Food consumption was not allowed during the dialysis procedure and all participants was wearing standardized clothing to secure the same insulating properties of clothing during the different dialysis protocols. During the different dialysis protocols, core temperature (T_C_) and mean skin temperature (T_sk_) were recorded. The body heat storage (S) for every time point (minute) was calculated during all scenarios using the thermometric method published by Burton [23]. The data recording lasted 5 h for each patient (1 h before dialysis protocol and 4 h during the dialysis protocol). The exercise program during TD + E and CD + E was performed between the 60th and the 120th minute of the dialysis protocol.

### Core temperature measurements (T_c_)

Core temperature (T_c_) was measured at the gastrointestinal tract using an ingestible telemetric pill (CorTemp, Human Technologies, Inc., St. Petersburg, USA). Data recorded continuously at 1-min intervals, throughout the course of the experimental intervention. The telemetric pill was ingested by the patients 7-h before arriving in the hospital [24, 25].

### Mean skin temperature measurements (T_sk_)

Skin temperature was measured at 1-min intervals by wireless iButtons (iButton, Maxim, USA). The iButtons were programmed before their application on the skin, as outlined by the manufacturer. The iButton resolution was fixed at 0.06 °C and the iButton clock was matched with a PC. The iButtons were attached on the skin using water-resistant, medical-grade tape. In total four iButtons were attached on the skin, at the following anatomic locations: on the biceps, pectoralis major, rectus femoris, and gastrocnemius, and were used to calculate mean skin temperature (T_sk_) using Ramanathan equation [23].

### Body heat storage (S)

Body heat storage for every minute was calculated during all conditions using the thermometric method proposed by Burton: *S = 3.47· m*_*b*_ *· ΔṪb* where 3.47 is the average specific heat of body tissues (in kJ·kg^− 1^·°C^− 1^), *m*_b_ is the patient’s body mass (in kg), and ΔṪb is the rate of change in mean body temperature (Ṫb) at time t t from the beginning of HD (initial Ṫb at time 0) [26].

### Intradialytic exercise program

The patients performed cycling for 60 min in the supine position during the TD + EP and CD + EP protocols. Patients were asked to pedal on a bedside cycle ergometer (Model 881 Monark Rehab Trainer, Monark Exercise AB, Varberg, Sweden) at 60% of their pre-assessed maximum power capacity. The exercise regime started 1 h after the commencement of the hemodialysis session. The patients’ maximum power capacity was determined by a modified version of the Åstrand Bicycle Ergometer Test protocol at bedside on a previous dialysis session during hemodialysis. Exercise was well tolerated by all patients, and no adverse reactions were reported.

### Statistical analysis

A Multivariate Analysis of Variance (MANOVA) followed by post-hoc paired-samples t tests were used to assess the effects of time (− 30, 0, 30, 60, 90, 120, 150, 180, 210, 240) and protocol (TD, CD, TD + E, CD + E) on T_C_, T_sk_, and S. Based on published recommendations, [27, 28] the level of significance was not adjusted for the multiple comparisons conducted in these post hoc tests. The MANOVA results demonstrated no statistically significant main or interaction effects. However, the observed power in these tests ranged between 0.17 and 0.67 (with the exception of the protocol main effect in T_C_ which was 0.96). Given the very low power of these tests, the analysis was focused exclusively in the post-hoc paired-samples t tests. To further strengthen the analysis, Cohen’s d effect sizes (0.2–0.5: small effect; 0.5–0.8: moderate effect; > 0.80: large effect) were also used to identify paired differences between protocols and times. To increase the statistical power of the analysis, we also used a linear mixed effects model with hourly data. Data are expressed in mean ± SD. A *p* value < 0.05 was considered statistically significant. All analyses were carried out using the Statistical Package SPSS 21.

## Results

Ten stable chronic hemodialysis patients were eligible and consent to participated in the study. *A*ll participants completed all 4 dialysis protocols on a random order. The patient’s characteristics are presented in Table 1.

### Changes in Core body temperature (Tc) under four dialysis protocols

**Fig. 1:**
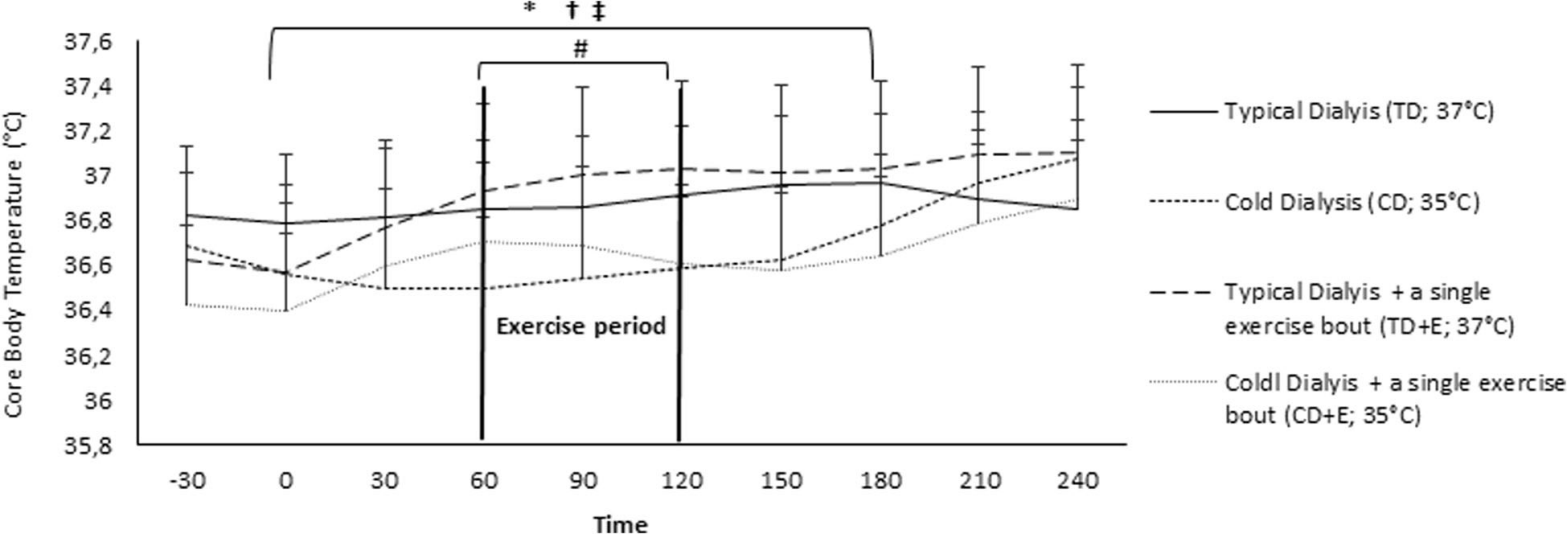
Core Body temperature (Tc) during 4 different dialysis protocols. *Statistically significant differences between TD vs CD protocol (*p* < 0.05). †‡. † Statistically significant differences between TD vs CD + E protocol. ‡ Statistically significant differences between TD + E vs CD protocol. # Statistically significant differences between CD + E vs CD protocol during exercise period. Abbreviations: TD, typical dialysis; CD, cold dialysis; E, exercise

The TD protocol resulted in an increase of core body temperature (T_C_) compared to the CD protocol (TD: 36.9 ± 0.1 °C; CD: 36.7 ± 0.2 °C). This was evident by statistically significant differences (*p* < 0.05) and moderate or large effect sizes between CD and TD from 0 to 180 min (Fig. 1).

However, the T_C_ remained similar during the TD + E and the TD protocols (TD: 36.9 ± 0.1 °C; TD + E: 36.9 ± 0.2 °C) except during the period that exercise training took place, where a slight increase in T_C_ was evident (TD: 36.9 ± 0.1 °C; TD + E: 37.0 ± 0.1 °C) resulting in a medium effect size observed at the end of exercise (Fig. 1).

The T_C_ during the CD + E protocol was lower than during the TD (TD: 36.9 ± 0.1 °C; CD + E: 36.6 ± 0.2 °C), which was evident by significant reductions (*p* < 0.05) and moderate or large effect sizes until 180 min into the protocol (Fig. 1). These differences were also observed during the exercise period of the CD + E (TD: 36.9 ± 0.1 °C; CD + E: 36.7 ± 0.1 °C).

The T_C_ increased almost throughout the TD + E protocol compared to the CD (CD: 36.7 ± 0.2 °C; TD + E: 36.9 ± 0.2 °C), which was evident by statistically significant differences (*p* < 0.05) and moderate or large effect sizes (Fig. 1).

During the exercise period of the CD + E protocol (CD: 36.5 ± 0.1 °C; CD + E: 36.7 ± 0.1 °C), the T_C_ was significantly increased compared to the CD (*p* < 0.05 and moderate effect sizes from 60 to 90 min), yet the low dialysate temperature used in the CD + E was able to disseminate this additional amount of heat (Fig. 1). As a result, the T_C_ was similar across the CD and the CD + E protocols (CD: 36.7 ± 0.2 °C; CD + E: 36.6 ± 0.2 °C).

### Changes in mean skin temperature (tsk) under four dialysis protocols

**Fig. 2:**
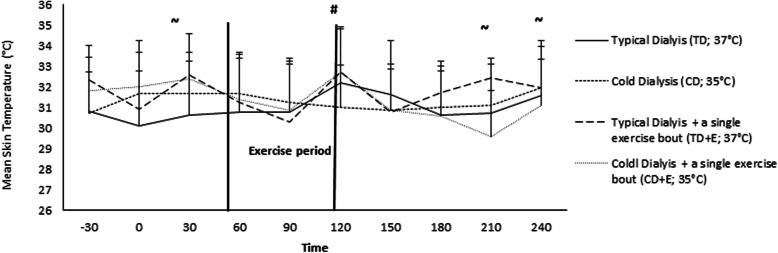
Mean skin temperature (Tsk) during 4 different dialysis protocols. ~ Moderate effect size (d:50–80) at time points 30, 210 and 240 min between TD vs TD + E and CD vs TD + E protocols. # Statistical significant differences between CD + E vs CD protocol at time point 120 min. Abbreviations: TD, typical dialysis; CD, cold dialysis; E, exercise

The mean skin temperature (T_sk_) were similar during TD and CD protocols (TD: 31.0 ± 0.6 °C; CD: 31.3 ± 0.4 °C; *p* > 0.05) as well as between TD and CD + E (TD: 31.0 ± 0.6 °C; CD + E: 31.3 ± 0.9 °C; *p* > 0.05). However, the T_sk_ was increased during the TD + E compared with the TD protocol (TD: 31.0 ± 0.6 °C; TD + E: 31.7 ± 0.8 °C) resulting in moderate effect sizes observed at 30, 210, and 240 min in the protocol (Fig. 2). In addition, the TD + E protocol resulted in somewhat increased in T_sk_ compared with CD (CD: 31.3 ± 0.4 °C; TD + E: 31.7 ± 0.8 °C) which was indicated via moderate effect sizes at different points (i.e., 30, 120, and 210 min) during the protocol.

Also, T_sk_ slightly increased during the exercise period of the CD + E protocol compared with the CD protocol (CD: 31.3 ± 0.4 °C; CD + E: 31.7 ± 0.9 °C), paralleled with a *p* value of 0.08 and a moderate effect size at 120 (Fig. 2). However, there was no overall T_sk_ differences between the CD and the CD + E protocols (CD: 31.3 ± 0.4 °C; CD + E: 31.3 ± 0.9 °C). Regarding comparison between TD + E protocol and CD + E resulted in somewhat increased T_sk_ (TD + E: 31.7 ± 0.8 °C; CD + E: 31.3 ± 0.9 °C; (Fig. 2).

### Changes in body heat storage (S) under four dialysis protocols

**Fig. 3:**
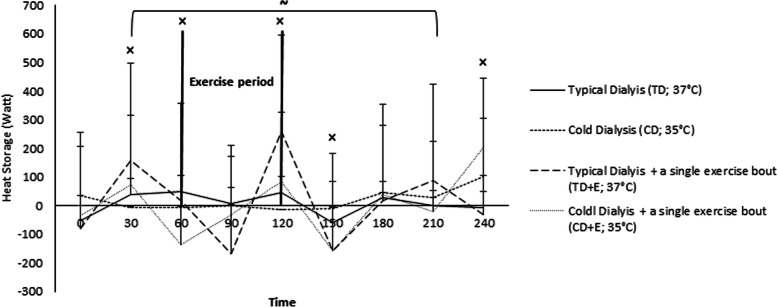
Body heat storage (S) during 4 different dialysis protocols. ~ Moderate effect size (d:50–80) at time points 30–210 min between TD vs TD + E and CD vs TD + E protocols. × Statistically significant differences between CD + E vs CD protocol at time point 30, 60, 120, 150 and 240 min

The body heat storage (S) was similar during the TD and CD protocol (TD: 5.5 ± 40.5 W; CD: 19.3 ± 37.7 W; *p* > 0.05 and d < 0.4). Overall, the S was slightly increased during the TD + E protocol (TD: 5.5 ± 40.5 W; TD + E: 11.2 ± 141.4 W) and particularly during the exercise period, as indicated by moderate effect sizes compared with TD (Fig. 3). Additionally, the S was similar between TD and CD + E (TD: 5.5 ± 40.5 W; CD + E: 2.2 ± 112.5 W; *p* > 0.05 and d < 0.4). During the TD + E, the S was somewhat increased compared to CD (CD: 19.3 ± 37.7 W; TD + E: 11.2 ± 141.4 W), observed via moderate effect sizes at different points (i.e., 30, 150, and 240 min) during the protocol. The slight increases in T_C_ and T_sk_ during the CD + E were also evident in terms of S, where moderate/large effect sizes were observed at different time points (i.e., 30, 60, 120, 150, and 240 min) compared with the CD. However, as a whole, there were no major differences in S between the two protocols (CD: 19.3 ± 37.7 W; CD + E: 2.2 ± 112.5 W). In terms of S, the CD + E led to attenuated values compared to TD + E, particularly during the exercise phase, as evidenced by moderate effect sizes (Fig. 3).

### Patients’ symptoms and side effects under the four dialysis protocols

Participants tolerated all four protocols with no major complains. The most common symptom during CD was the sensation of “goose bumps” that it was addressed by covering the patient’s body with a light sheet. Similarly, during exercise protocol, patients complained for a mild muscle burning sensation during the first 5 min of the bout which disappeared after the completion of the warm up period. No major adverse effects reported during the course of the study.

## Discussion

The present study, sought to examine for the first time the separate and combined effects of cold dialysis and intradialytic exercise training on the thermoregulatory responses (core temperature, skin temperature, and body heat storage) of stable hemodialysis patients using for the first-time whole-body direct temperature assessment tools. Our results demonstrated that the TD and TD + E protocols are associated with increased body heat storage leading to moderate increases in core temperature (as high as 0.4 °C). Some studies have reported that, such changes in body heat storage and core temperature could cause peripheral vasodilation [29, 30] and may offset the vasoconstrictive response to hypovolemia [7]. Some data indicate that intradialytic hypotension can be attributed, among others, to hypovolemia; however, other data did not confirm such hypothesis [31]. Intradialytic hypotension has been associated with patient discomfort and increased mortality [8, 9]. Still, the above hypotheses have not been fully confirmed and thus, further studies are needed to clarify those issues. Therefore, the present detailed thermoregulatory assessment results confirm previous evidence suggesting that TD represents a challenge for the thermoregulatory system of patients with end-stage renal disease especially in developing countries where dialysis units ambient conditions are inadequately controlled [1, 2]. In contrast, the low temperature of the dialysate during the CD and the CD + E protocols prevented the rise in body heat storage and core temperature, even during the period that exercise training took place.

**Fig. 4:**
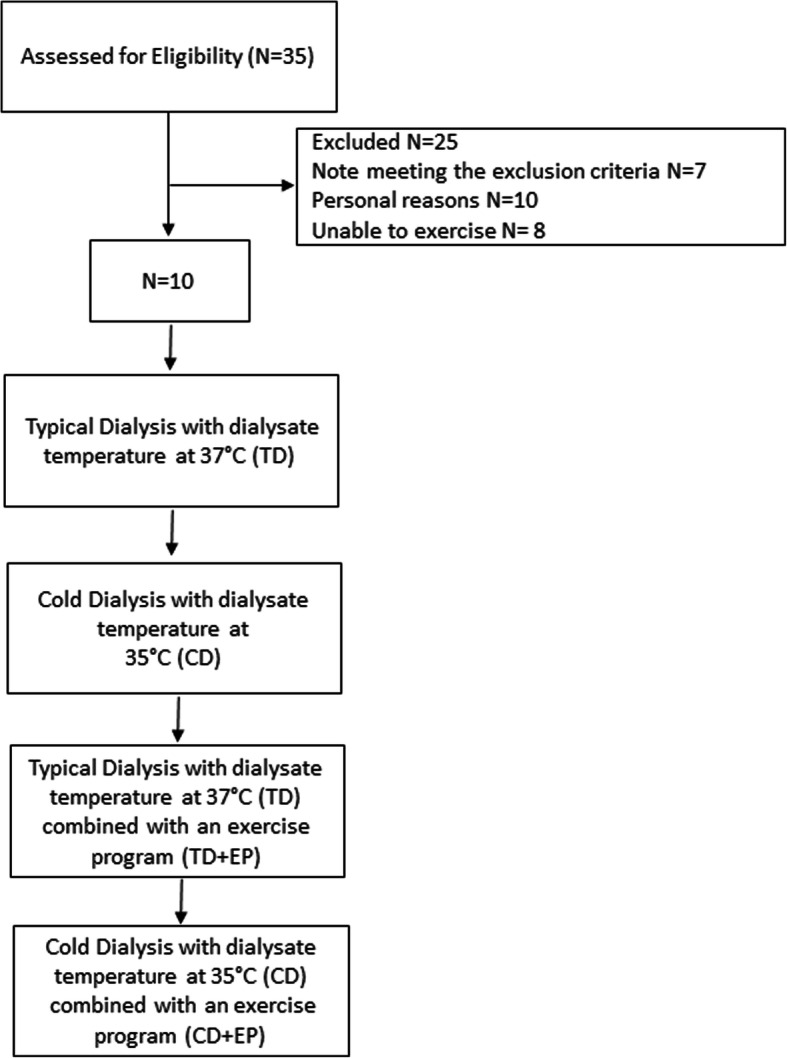
Flow chat of the study

It has been well-established that intra-dialytic exercise leads to benefits in physical performance and quality of life in hemodialysis patient [14–16]. Our results demonstrated that during exercise phase and especially when the dialysis temperature was at 37 °C, body heat storage slightly increased (Fig. 4). Studies show that during the intradialytic exercise an increased in skin blood flow limits the cardiovascular adjustment needed for work, because skin circulation participated both in hemodynamic control and thermoregulation [14].

Even though the uniqueness of our study as well as the laborious methodology and highly skilled personnel required a number of limitations unfortunately still remain. It is important to denote that we were unable to obtain peripheral/skin blood flow data assessing the blood redistribution occurred during the 4 different scenarios, energy expenditure during exercise as well as the blood temperature that is available on the dialysis machines’ monitors. Therefore, our inferences regarding peripheral vasoconstriction/vasodilation stem from skin temperature measurements. Nevertheless, it is well known that skin temperature is very well correlated with changes in the cutaneous circulation [29, 30]. Another issue to consider is the effect of fixed reduction in dialysate temperature. As a consequence, the dialysis treatment must be adapted to the patient’s individual condition and response to treatment. Indeed, recent reports support the link between cold dialysis and low intradialytic hypotension episodes in the hypotensive HD patients [32].

However, the used of ingestible telemetric pill (temperature sensor) represents a valid index of body core temperature (Tc) that is convenient and shows excellent utility for prove valuable in field studies, in investigations requiring frequent measurements over long periods, or if the subject needs to be entirely free during the observations [25].

## Conclusions

In conclusion, the results of the present study demonstrate that typical dialysis and intradialytic exercise are accompanied by a moderate level of hyperthermia, which can be offset by cold dialysis. Based on these findings, we observed that hemodialysis sessions which incorporate cold dialysis alone or supplemented with intradialytic exercise can prevent the rise of body heat storage. The latter has been shown to be a major factor for developing intradialytic hypotension, which is due to both hemodynamic responses (hypovolaemia stress) and thermoregulatory responses (thermal stress) during hemodialysis. However, further studies are needed to determine the above mechanisms both during cold dialysis and intradialytic exercise for the prevention of hyperthermia during dialysis.

## Data Availability

The datasets of the current study are available from the corresponding author on reasonable request.

## Abbreviations

IET: Intradialytic exercise training
CD: Cold dialysis
TD: Typical dialysis
S: Body heat storage
HD: Hemodialysis
T_sk_: Mean skin temperature
*m*_b_: Patient’s body mass (in kg)
ΔṪb: The rate of change in mean body temperature
Ṫb: Mean body temperature
MANOVA: Multivariate Analysis of Variance
SD: Standard deviation
